# Novel variants impairing Sp1 transcription factor binding in the *COL7A1* promoter cause mild cases of recessive dystrophic epidermolysis bullosa

**DOI:** 10.1038/s41431-024-01717-5

**Published:** 2024-12-05

**Authors:** Nathalie Pironon, Artyom Gasparyan, María Joao Yubero, Sabine Duchatelet, Kristina Hovhannesyan, Stephanie Leclerc-Mercier, Natella Kostandyan, Francis Palisson, Tamara Sarkisian, Matthias Titeux, Ignacia Fuentes, Alain Hovnanian

**Affiliations:** 1 Laboratory of Genetic Skin Diseases, Institut Imagine, Université Paris Cité, Inserm, UMR 1163, F-75015 Paris, France; 2 Center of Medical Genetics and Primary Health Care, Abovyan Street, Yerevan, Armenia; 3 Facultad de Medicina, Clínica Alemana Universidad del Desarrollo, Santiago, Chile; 4 DEBRA Chile, Francisco de Villagra 392, Ñuñoa, Santiago, Chile; 5 Department of Pathology, AP-HP, Hôpital Necker-Enfants Malades, F-75015 Paris, France; 6 Centro de Genética y Genómica, Facultad de Medicina, Clínica Alemana Universidad del Desarrollo, Santiago, Chile; 7 Departamento de Biología Celular y Molecular, Facultad de Ciencias Biológicas, Pontificia Universidad Católica de Chile, Santiago, Chile; 8 Department of Genomic Medicine of Rare Diseases, AP-HP, Hôpital Necker-Enfants Malades, F-75015 Paris, France

**Keywords:** Medical genetics, Diseases

## Abstract

Recessive dystrophic epidermolysis bullosa (RDEB) is a rare and most often severe genodermatosis characterized by recurrent blistering and erosions of the skin and mucous membranes after minor trauma, leading to major local and systemic complications. RDEB is caused by loss-of-function mutations in *COL7A1* encoding type VII collagen (C7), the main component of anchoring fibrils which form attachment structures stabilizing the cutaneous basement membrane zone. Most of the previously reported *COL7A1* mutations are located in the coding or intronic regions. We describe 6 patients with localized or intermediate RDEB for whom one recessive pathogenic variant in the coding region and a second variant in the *COL7A1* promoter were identified. These substitutions, three of which are novel, are localized in two Sp1 binding sites of the promoter region. DNA pull-down assay showed a drastic reduction of Sp1 binding consistent with a dramatic decrease in *COL7A1* transcript and almost undetectable C7 protein levels. Our results reveal that mutations in the *COL7A1* promoter on the background of a null allele can underlie localized or intermediate RDEB. They further emphasize the functional importance of Sp1 motifs in the proximal *COL7A1* promoter which should be carefully investigated for regulatory mutations in the case of RDEB with only one pathogenic variant identified in the coding or intronic regions.

## Introduction

Dystrophic epidermolysis bullosa (DEB) is a rare blistering disorder caused by mutations in the *COL7A1* gene encoding type VII collagen, a major component of anchoring fibrils [1]. DEB can be inherited in an autosomal dominant or recessive pattern. To date, approximately 1200 distinct *COL7A1* variants have been reported (Human Genome Mutation Database). Around 43% of mutations are missense mutations, 19% are splicing mutations, 22% are small out-of-frame insertions or deletions and 10% are nonsense mutation. Additional rare mutations include regulatory mutations, gross insertion or deletion, small indels and complex rearrangement. Only 2 regulatory mutations have been reported in the *COL7A1* promoter (c.-187C > T and c.-188C > T) in 2 patients with severe RDEB. These mutations affected the same binding site of the transcription factor Sp1, and were shown significantly reduce gene expression [1, 2].

Here, we describe six unrelated patients with localized or intermediate recessive DEB whose second *COL7A1* mutation could not be detected in the coding sequences but was identified within the *COL7A1* promoter region which carried 4 distinct mutations, 3 of which being new variants.

## Materials and methods

### EB cases

The patients included in this study were referred to us for the diagnosis of RDEB on the basis on their clinical presentation. After informed consent was signed, blood samples were taken from the index cases and their parents when available, and a skin biopsy was taken for diagnostic purpose (and for the father of patient 1).

### Immunofluorescence staining

Immunofluorescence staining for type VII collagen was performed to explore the consequences of *COL7A1* mutations on type VII collagen expression. Five µM frozen skin sections were fixed in acetone at −20 °C for 10 min. Type VII collagen staining was performed using the mouse monoclonal LH7.2 primary antibody, 2 h at room temperature at 1:2000 (Sigma-Aldrich, Saint Quentin Fallavier, FR; C6805), then incubated for 1 h with the Alexa Fluor 488 goat anti-mouse secondary antibody (1:200; Thermo Fisher Scientific, Villebon-sur-Yvette, FR). Mowiol mounting medium containing DAPI (0.5 µg/mL) was used. Images were captured with a Leica TCS SP8 confocal microscope.

### Transmission electron microscopy

A skin biopsy, from a clinically unblistered area, was immersed in 2.5% glutaraldehyde fixative in 0.1 M cacodylate buffer at pH 7.4 for 3–5 h at +4 °C, then washed thoroughly in cacodylate buffer overnight at +4 °C and postfixed in 1% osmium tetroxide for 1 h at room temperature. The skin biopsy slices were then dehydrated in graded ethanol and impregnated with epoxy resin. Semithin sections revealed the presence of cleaved and uncleaved areas. They were stained with 1% toluidine blue and examined with a light microscopy. Ultrathin sections were performed and stained with uranyl acetate followed by lead citrate for examination by electron microscopy.

### DNA analyses

Mutational analysis of *COL7A1* was performed by Next-Generation Sequencing using a custom panel (which includes the promoter sequence) and confirmed by Sanger sequencing. In silico prediction of the consequences of the *COL7A1* promoter mutations on the binding of transcription factors was made using the JASPAR, ALGGEN-PROMO, ALIBABA2 and TFBIND programs retrieved from http://jaspar.genereg.net/, http://alggen.lsi.upc.es/, http://gene-regulation.com/pub/programs/alibaba2/, and https://tfbind.hgc.jp/, respectively.

### DNA fragment analysis by capillary electrophoresis

For GeneScan analysis, the 5’-end of the forward PCR primer for *COL7A1* was labeled with FAM fluorophore. The purified amplified products were diluted with internal lane standards GeneScan™ 500 ROX™ dye Size Standard (Applied Biosystems, Waltham, MA) and highly deionized-formamide (Applied Biosystems). After shaking, the reaction system was heated at 95 °C for 5 min, then rapidly placed on ice for 5 min. The mixtures were scanned by capillary electrophoresis method using an ABI 3500XL Gene Analyzer (Applied Biosystems). The GeneMapper 5 software (Applied Biosystems) was used for the analysis of capillary electrophoresis results. For patient 1, the following primers were used: 5′FAM-TGACCTGCACGCGCCTTTACGC-3′ (Exon 2) and 5′-CCACAGCAAATAGCTTGACCCC-3′ (Exon 4) and the control sample showed a single peak at 435 bp corresponding at the wt transcript. For patients 3 and 4.1, the following primers were used: 5′FAM-ACCCGGGTCTACCAGGAGAG-3′ (Exon 79) and 5′-TCTCCATGACCACCCACTG-3′ (Exon 81) and the control sample showed a single peak at 100 pb corresponding to the wt transcript.

### Fibroblast culture and expression studies

Primary fibroblasts were isolated from a skin biopsy for patients 1, 3 and 4.1 and from healthy individuals undergoing surgery and were cultured in DMEM medium (Thermo Fisher Scientific, Villebon-sur-Yvette, FR). The effects of *COL7A1* mutations on mRNA were analyzed by quantitative reverse transcriptase (RT)-PCR analysis of total RNA using MESA GREEN qPCR MasterMix Plus (Kaneka Eurogentec, Seraing, B). The following primers were used: 112 F 5′-AGAAGGGAGAAGCTGCACTG-3′ and 114/115 R 5′-CATAACTAGGGAGGGGTCGTGA-3′ for *COL7A1* and PGK-F 5′-CTGTGGCTTCTGGCATACCT-3′ and PGK-R 5′-AATCTGCTTAGCCCGAGTGA-3′ for *PGK* (Phosphoglycerate kinase). Type VII collagen protein levels were analyzed by immunoblotting of lysates of cultured fibroblasts after the addition of TGF-β (LH7.2 monoclonal antibody for C7). β-actin was used as loading control. Protein Quantification was performed by Bradford Assay (Bio-Rad, Marnes-la-Coquette, FR).

### DNA pull-down assay

For the DNA pull-down assay, the following single stranded biotinylated sense oligonucleotides and their complementary were used: 5′-GCCTGCCGCTCCGCCCCCCGAGAT-3′, corresponding to the *COL7A1* wild-type (wt) sequence from position −198 to −174; 5′-GCCTGCCGCTCCG**T**CCCCCGAGAT-3′, corresponding to the mutated sequence c.-185C > T; 5′-GCCTGCCGCTCCG**A**CCCCCGAGAT-3′, corresponding to the mutated sequence c.-185C > A; 5′-GCCTGCCGCTC**T**GCCCCCCGAGAT-3′, corresponding to the mutated sequence c.-187C > T ; 5′-TATGGGTGAGGGCGGGTGCCTGGG-3′, corresponding to the *COL7A1* wild-type sequence from position −227 to −203; 5′-TATGGGTGAGGG**T**GGGTGCCTGGG-3′, corresponding to the mutated sequence c.-215C > T and 5′-GCTCGCC**CCGCCC**CGATCGAAT-3′, the oligonucleotide containing the consensus sequence for Sp1 used by Gardella [2] as a control sequence. These oligonucleotides and their complementary strands were hybridized by incubation at 100 °C for 1 h and then allowed to cool down slowly (for approx. 30 min) to room temperature. Nuclear extracts (500 µg/samples) from Hela cells were mixed with biotinylated double-stranded oligonucleotides and incubated on a rotating shaker at +4° overnight. DNA-protein complexes were further incubated with streptavidin-agarose beads for 2 h at +4° on a shaking platform. Thereafter the samples were centrifuged at 550 g for 1 min and the pellet was washed 5 times in washing buffer (PBS, 1 mM EDTA, 1 mM DTT, protease inhibitor cocktail Complete (Sigma-Aldrich)). Finally the pellet was dissolved in 35 μl 2× Laemmli sample buffer, incubated at 100 °C for 5 min to detach the proteins bound to the oligonucleotides and centrifuged at 7000 g for 30 s. The supernatant was collected and loaded on a SDS-polyacrylamide gel to perform western-blot analysis using Sp1 antibodies.

## Results

**Fig. 1:**
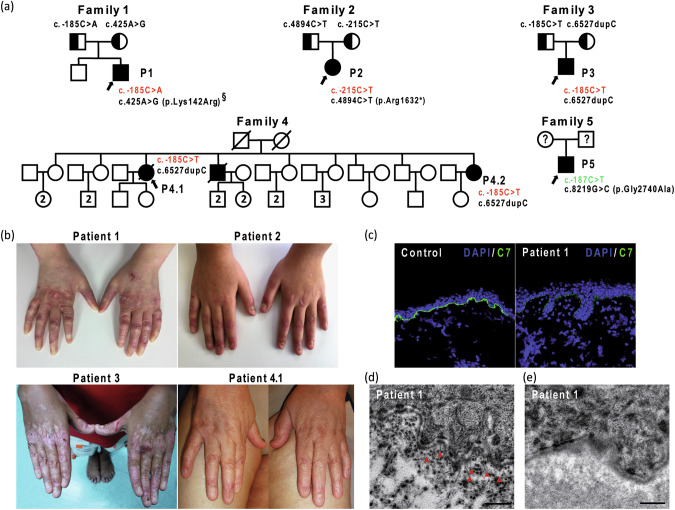
Clinical and molecular characterization of the probands. **a** Pedigree of the probands (The number inside the symbol corresponds to the number of siblings). Previously described mutations are depicted in green, new mutations reported in this study are in red. **b** Clinical presentations of the probands. **c** Immunofluorescence staining of patient 1 with LH7.2 monoclonal antibody showing drastically reduced and discontinuous C7 staining along the dermo-epidermal junction in comparison with strong and continuous staining in the control. **d** Electron microscopy of patient 1 showing numerous anchoring fibrils (red arrows) beneath the lamina densa in an uncleaved area. **e** Electron microscopy of patient 1 showing absence of anchoring fibrils beneath the lamina densa in a cleaved area (The light grey area corresponds to the blister cavity). Bar = 500 nm. (§This mutation is a previously described splicing mutation which inhibits normal splicing of exon 3 producing 3 abnormal transcripts with premature stop codons).

**Fig. 2:**
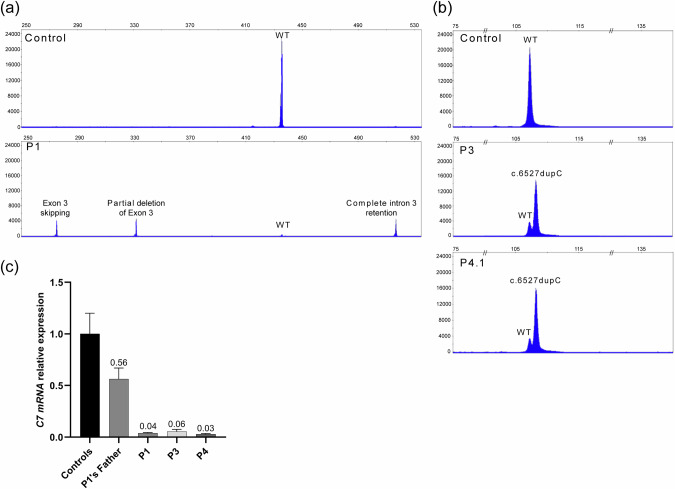
Molecular characterization of individuals 1, 3 and 4.1 with recessive dystrophic epidermolysis bullosa. **a**, **b** GeneScan RT-PCR analysis showing the presence of a small amount of wild-type transcript in the fibroblasts of patients 1, 3 and 4.1. (For figure b, the PCR products from patients 3 and 4.1 were less diluted than the control to show the presence of the wt transcript) (**c**) Quantitative RT-PCR analysis of *COL7A1* expression relative to *PGK* expression using primers amplifying exon 112 to exon 114 in *COL7A1*. *COL7A1* transcript levels are strongly reduced in fibroblasts of patients 1, 3 and 4.1. Experiments were replicated three times.

**Fig. 3:**
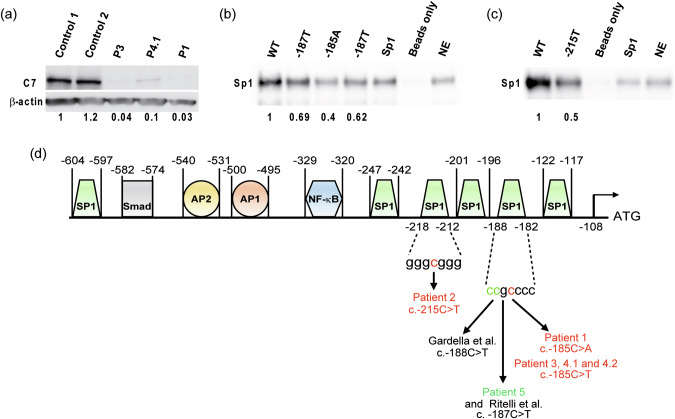
Western-Blot analysis and functional analysis of novel promoter variants. **a** Western-blot analysis show very low amount of wild-type C7 protein in fibroblasts from RDEB patients 1, 3 and 4.1. Data are representative of three independent experiments. **b**, **c** Pull-down assay of Sp1 binding to a WT or mutated sequence of the *COL7A1* promoter. HeLa Nuclear extracts (NE) were mixed with biotinylated DNA probes. The labeled DNA probes were precipitated with streptavidin-agarose beads, and precipitates were subjected to SDS-PAGE, followed by immunoblotting with anti-Sp1 antibody. Data are representative of three independent experiments. (Lane Sp1: Sp1 consensus sequence used; Lane NE: Nuclear extracts with no oligonucleotides are deposited on the gel to verify the presence of Sp1 in the nuclear extracts). **b** The mutated sequences c.-185T and c.-185A lead to reduced binding of the transcription factor Sp1 compared with the WT sequence (c.-185C). **c** The mutated sequence c.-215T shows decreased Sp1 binding compared to the WT sequence c.-215C. **d** Schematic representation of the *COL7A1* promoter with transcription factor binding sites and corresponding transcription factors indicated. Schematic based on Kon and Naso [20, 21]. Previously described mutations are depicted in green, new mutations reported in this study are in red.

Patient 1, a 10-year-old boy of French extraction, was born to non-consanguineous parents and had an unaffected brother. He presented with intermediate RDEB with limited digital fusion of fingers, blisters with prominence over hands, feet, elbows and knees, and no mucous membrane involvement (Fig. S1). He harbored the c.425 A > G (NM_000094.3) splicing mutation in exon 3 in *COL7A1* at the heterozygous state inherited from his mother (Fig. 1a). This recessive mutation has been previously reported [3, 4] and completely inhibits normal splicing of exon 3. As a result, 3 abnormal transcripts are produced resulting in premature stop codons arising from the retention of intron 3 (p.Cys144Ilefs*22), partial deletion of exon 3 due to the activation of an upstream exonic GT cryptic site (p.Glu109Leufs*36) and out-of-frame skipping of exon 3 (p.Thr90Serfs*4). In agreement with these reports, capillary electrophoresis analysis of RT-PCR products from patient 1’s keratinocytes detected these three abnormal transcripts (39, 30 and 26% of observed transcripts respectively) and low levels of normal *COL7A1* transcripts (5%) (Fig. 2a). No additional mutation was identified in the entire *COL7A1* coding sequence and no abnormal *COL7A1* transcript was detected by RT-PCR covering the entire *COL7A1* coding sequence. However, quantitative RT-PCR showed a ~ *2*-*fold* and *25-fold* reduction in *COL7A1* mRNA expression in the fibroblasts from the father and patient 1, respectively (Fig. 2c). These results suggested that the *COL7A1* mutation inherited from the father led to the absence or marked reduction of detectable *COL7A1* mRNA. As no mutation in the coding sequence was identified in patient 1, we carefully analyzed the promoter region of *COL7A1* and identified a regulatory mutation (NC_000003.11:g.48632777 G > T; NM_000094.3:c.-185C > A) in the same consensus sequence (ccg**c**cc) for the binding of the Sp1 transcription factor as two previously described *COL7A1* promoter mutations [2, 5]. This mutation was present at the heterozygote state in patient 1, was inherited from his father and was predicted to impair Sp1 binding by in silico prediction tools (JASPAR, ALIBABA2 and ALGGEN-PROMO). Western-blot analysis showed no detectable wild-type type VII collagen protein in patient 1’s cultured fibroblasts (Fig. 3a). In line with this result, type VII collagen Immunofluorescence staining was drastically reduced in patient 1’s skin (Fig. 1c). Electron microscopy analysis showed cleavage below the lamina densa with numerous and sometimes short anchoring fibrils in uncleaved areas (Fig. 1d) whereas anchoring fibrils were not detectable in cleaved skin areas (Fig. 1e).

Patient 2, a 12-year-old girl originating from Armenia presented with localized RDEB with skin blistering limited to hands and feet, occasionally to pretibial skin, with milia and loss or dystrophic finger and toe nails (Figs. 1b, S2). She carried a previously reported recessive nonsense p.Arg1632* (c.4894 C > T) mutation in exon 51 inherited from her unaffected father [6–8] (Fig. 1a) with no detectable second mutation in the coding sequence and flanking intronic regions in *COL7A1*. Similar to patient 1, analysis of the *COL7A1* promoter sequence revealed a new regulatory mutation (NC_000003.11:g.48632807 G > A; NM_000094.3:c.-215C > T) inherited from her unaffected mother. The c.-215C > T substitution is located in a Sp1 binding sequence located upstream of the Sp1 binding site disrupted by mutation c.-185C > A) (Fig. 3d) and was predicted to impair Sp1 binding by in silico prediction tools (JASPAR, TFBIND, ALIBABA and ALGGEN-PROMO).

Patient 3 is a 22-year-old man originating from Chile. He is affected with intermediate RDEB with predominantly blistering on hands, feet, elbows and knees, loss of finger and toe nails, no mucosal lesion and no digital fusion (Figs. 1b, S3).

Patient 4.1, is a 70-year-old female (at the time of biopsy) presenting with intermediate RDEB, originating from Chile, unrelated to patient 3. She was born to healthy parents and had two siblings also affected with RDEB, one of whom died from pulmonary fibrosis at the age of 63. She had localized RDEB with skin blistering limited to acral sites and to pretibial skin, with loss of finger and toe nails (Fig. 1b). Patient 4.1 died at the age of 73 years from pulmonary fibrosis. Her youngest sister (P4.2) is 55 year-old and is affected with a similar clinical phenotype consistent with intermediate RDEB.

Patients 3, 4.1 and 4.2 were heterozygotes for a Spanish recessive founder frameshift p.Gly2177Trpfs*113 (c.6527dupC) mutation in exon 80 of *COL7A1* with no additional variation identified in the coding region and flanking intronic regions of *COL7A1*. Examination of the promoter region disclosed a new regulatory mutation NC_000003.11:g.48632777 G > A; NM_000094.3:c.-185C > T changing the same nucleotide position as patient 1 (but not the same substitution) in the previously reported Sp1 binding site (ccg**c**cc) [2, 3, 9–13]. The c.-185C > T mutation was predicted to impair Sp1 binding by in silico prediction tools (JASPAR, ALIBABA2 and ALGGEN-PROMO). Previous studies showed that the c.6527dupC mutation results in the absence of *COL7A1* mRNA and C7 protein [9–11]. qRT-PCR analysis showed a ~ *25*-*fold reduction* in *COL7A1* mRNA expression in fibroblasts from RDEB patients 3 and 4.1 (Fig. 2c). Capillary electrophoresis analysis of RT-PCR products from patient 3 and patient 4.1’s fibroblasts detected an abnormal transcript harboring the c.6527dupC mutation and low levels of the normal transcript (Fig. 2b). Western-blot analysis showed no detectable or very low amounts of wild-type type VII collagen protein in patient 3 and in patient 4.1’s fibroblasts, respectively (Fig. 3a).

Patient 5 is a 15-year-old teenager originating from France affected with intermediate RDEB. He was a heterozygote for the previously reported recessive *COL7A1* missense mutation p.Gly2740Ala (c.8219 G > C) in exon 110 [8]. In the absence of a secondary mutation in the coding and intronic sequences of *COL7A1*, analysis of the promoter sequence revealed the previously regulatory mutation NC_000003.11:g.48632779 G > A; NM_000094.3: c.-187C > T [5]. In contrast to the previously described c.-188C > T mutation, this change has been proposed to result in a significant reduction but not complete abrogation of transcription [2].

To ascertain the effect of these mutations on the binding of the transcription factor Sp1, we performed pull-down assays using biotinylated primers containing the wild-type or the mutated Sp1 sequences. The specificity of DNA-Sp1 interaction was attested by the binding of the Sp1 protein with a consensus Sp1 binding site sequence used by Gardella [2] (5′-GCTCGCC**CCGCCC**CGATCGAAT-3′). These results demonstrated that the wild-type sequences between −182 and −188, and between −212 and −218 bind the Sp1 protein, and that the c.-185C > A (patient 1), c.-185C > T (patients 3, 4.1 and 4.2), c.-187C > T (patient 5) and c.-215C > T (patient 2) transitions reduce the affinity of these sites for the Sp1 transcription factor (Fig. 3b, c).

## Discussion

We report the characterization of three novel mutations in the *COL7A1* promoter in patients affected with localized or intermediate RDEB. Only 2 mutations in the regulatory region have been reported so far. Gardella [2] reported the first mutation, the c.-188C > T substitution, in a patient presenting with severe RDEB. The authors showed by gel shift analysis that the −188/−182 sequence is a functional Sp1 site and that the -188C > T transition completely abrogates binding of the transcription factor. In 2013, Ritelli [5] identified a second mutation, c.-187C > T in the same sequence of the *COL7A1* regulatory region in a patient with severe RDEB, and concluded that this mutation drastically reduced but did not abolish the expression of the mutated allele. In our study, we report 2 novel mutations in the same −188/−182 functional Sp1 site, and 1 novel mutation in a novel −218/−212 functional Sp1 site in 6 patients with localized or intermediate RDEB (Fig. 3d). By DNA pull-down assay, we demonstrate that the 3 mutations affect binding of Sp1 to these sites, which is consistent with reduced *COL7A1* transcript levels as seen by qRT-PCR in patients 1, 3 and 4.1.

Sp1 is a transcription factor which regulates the expression level of several genes and mutations in the Sp1 binding consensus sequence have been reported to decrease the level of gene expression in several hereditary diseases [14, 15]. In particular, the Sp1 factor was shown to play a predominant role in the transcription of TATA-less genes, usually containing multiple Sp1 binding sites proximal to the +1 start site which can synergistically activate gene transcription [16]. The *COL7A1* promoter does not have CAAT nor TATA boxes and contains many potential Sp1 sites proximal to the transcription start. Vindevoghel [17] identified several Sp1 sites and demonstrated that one of them, positioned between residues −604 and −598, is necessary for the high basal expression of *COL7A1* in cultured keratinocytes and skin fibroblasts. Deletion or disruption of this site reduces by ~70–80% but does not abolish *COL7A1* expression levels. Moreover, Sasaki [18] also demonstrated that the GC-box located between nucleotides −247 and −242 is an important regulatory sequence since point mutations within this region markedly reduced Sp1 binding, identifying this site as crucial for high basal activity of the *COL7A1* promoter.

Our results indicate that the −188/−182 site and the −218/−212 site are also crucial Sp1 sites and are strong *COL7A1* regulatory elements whose integrity is necessary for basal expression of *COL7A1*. We anticipate that Sp1 site mutations could become therapeutic targets for ex vivo gene editing aiming at correcting these nucleotide sequences to restore normal Sp1 binding allowing increased *COL7A1* transcript levels. Of note, the promoter region of *COL7A1* contains two additional Sp1 binding sequences whose functional importance is currently not known. The functional results indicate that several Sp1 regulatory sequences contribute to the regulation of *COL7A1* transcription and that defects in only one of these sequences have the potential to drastically reduce or abolish *COL7A1* expression.

Since Sp1 sites within the *COL7A1* promoter play a crucial role in gene expression, it becomes essential to analyze these sites when a second mutation cannot be identified in the coding or intronic sequences of a patient with RDEB.

Genotype/phenotype correlation studies in RDEB have shown that the complete absence of C7 protein is most often associated with severe RDEB. It is striking to note that in contrast to two cases with severe (generalized) RDEB previously reported with a mutation in the *COL7A1* promoter region [2, 5], the phenotype of patients 1, 3, and 4.1 is only moderate despite barely detectable C7 protein (Immunofluorescence staining and/or western blot) and *COL7A1* mRNA transcripts. This cannot be explained solely by the nature of the second mutation since the second variant leads to PTC in these patients. It is possible that in vivo, the mutated promoter allows for residual expression of wild-type C7 protein during physiological regulation through other transcription factors binding sites present in the proximal promoter thus limiting skin fragility. In particular, binding sites for TGF-β and NF-κb are present in the proximal promoter of *COL7A1* (Fig. 3d). We can speculate that the very weak basal expression of C7 protein induces blisters, which trigger the activation of TGF-β and NF-κb pathways during wound healing, leading to the activation of the *COL7A1* promoter and the synthesis of wild-type C7 protein, thus limiting the extend and severity of the blisters and preventing chronic wounds. This possibility is supported by the fact that weak C7 protein labelling and numerous anchoring fibrils are seen in uncleaved skin of patient 1. It is also possible that differences in the expression of modifier genes such as *DCN* [19], epigenetic or environmental factors could contribute to the moderate phenotypes of these patients.

Our findings suggest that mutations in the *COL7A1* promoter, even when coupled with a mutation leading to a PTC, do not always lead to a severe RDEB phenotype. These mutations may instead manifest as localized or intermediate RDEB, as reported in our study. Consequently, thorough investigation of mutations affecting Sp1 and other transcription factor binding sites in the *COL7A1* promoter region is warranted in RDEB patients across the spectrum of severity, particularly when only one pathogenic recessive mutation was identified.

## Supplementary information

Figure S1

Figure S2

Figure S3

Supplementary materials

## Data Availability

All relevant data is available in the manuscript or supplement.
