# Supplementary figures and images for: Novel variants impairing Sp1 transcription factor binding in the COL7A1 promoter cause mild cases of recessive dystrophic epidermolysis bullosa
Source: Eur J Hum Genet. 2024 Dec 5;33(3):344–50. doi: 10.1038/s41431-024-01717-5 (PMC11894107; doi:10.1038/s41431-024-01717-5)

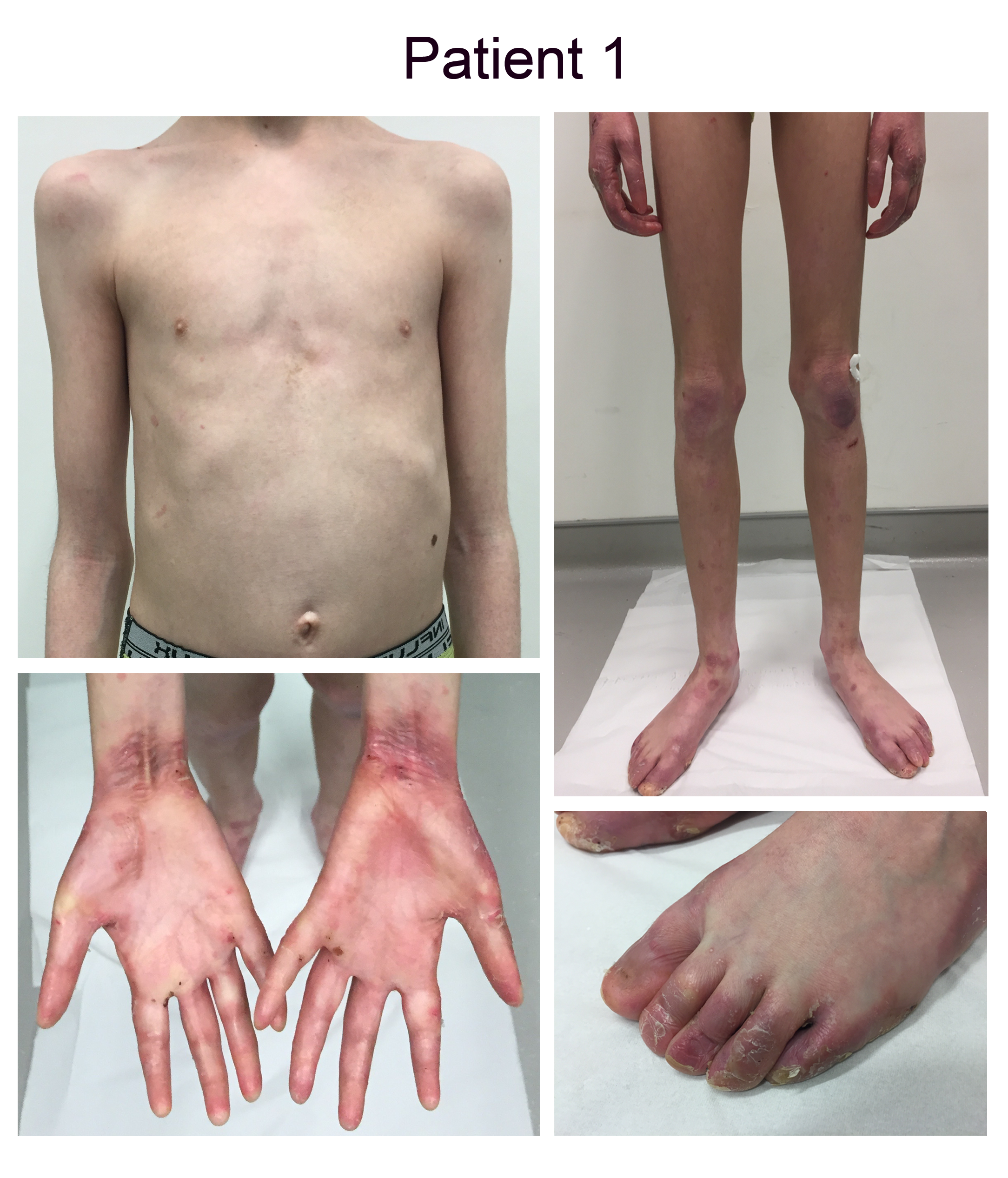

Supplement: Supplementary file 1 — Figure S1 [file 41431_2024_1717_MOESM1_ESM.tif]

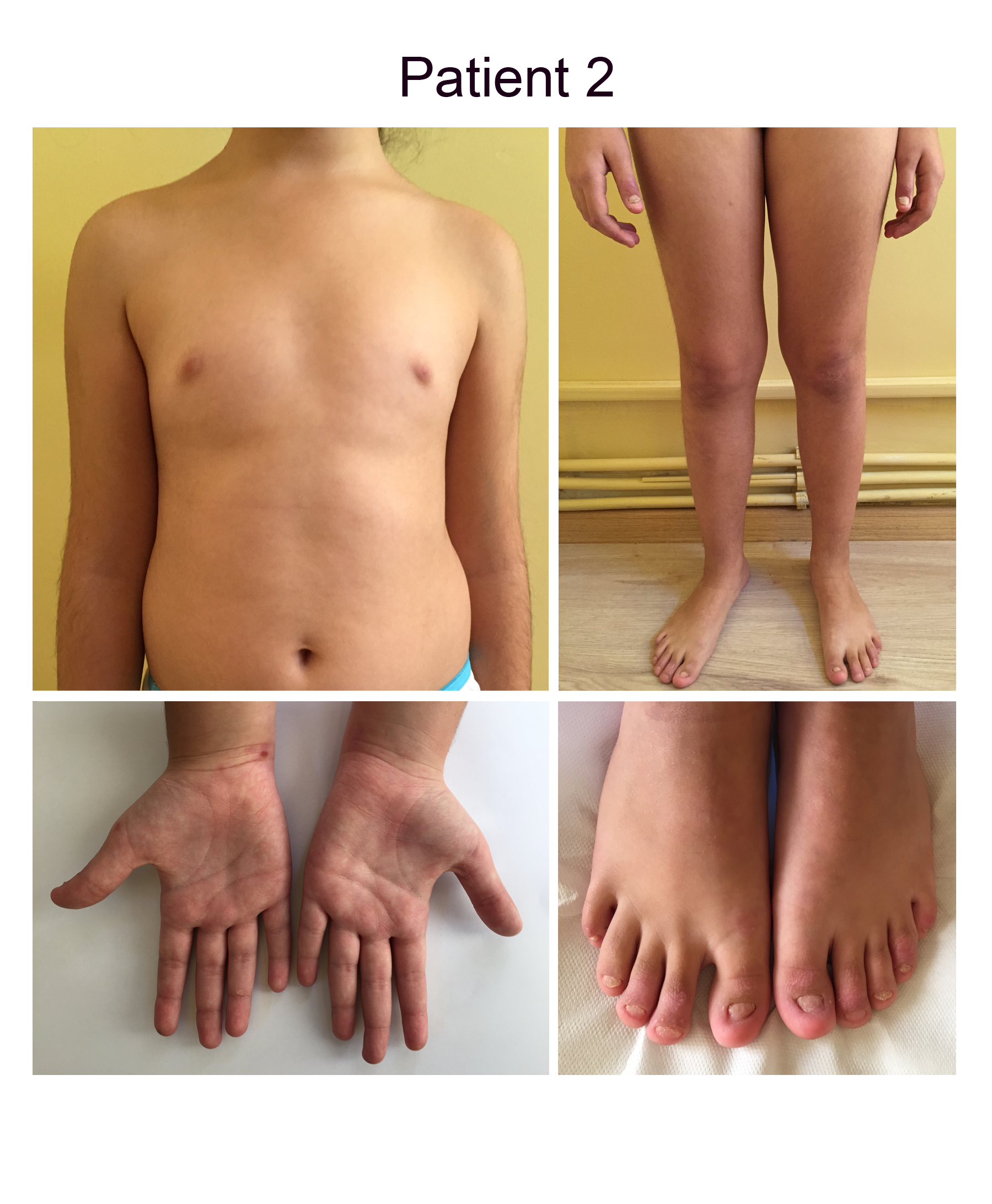

Supplement: Supplementary file 2 — Figure S2 [file 41431_2024_1717_MOESM2_ESM.tif]

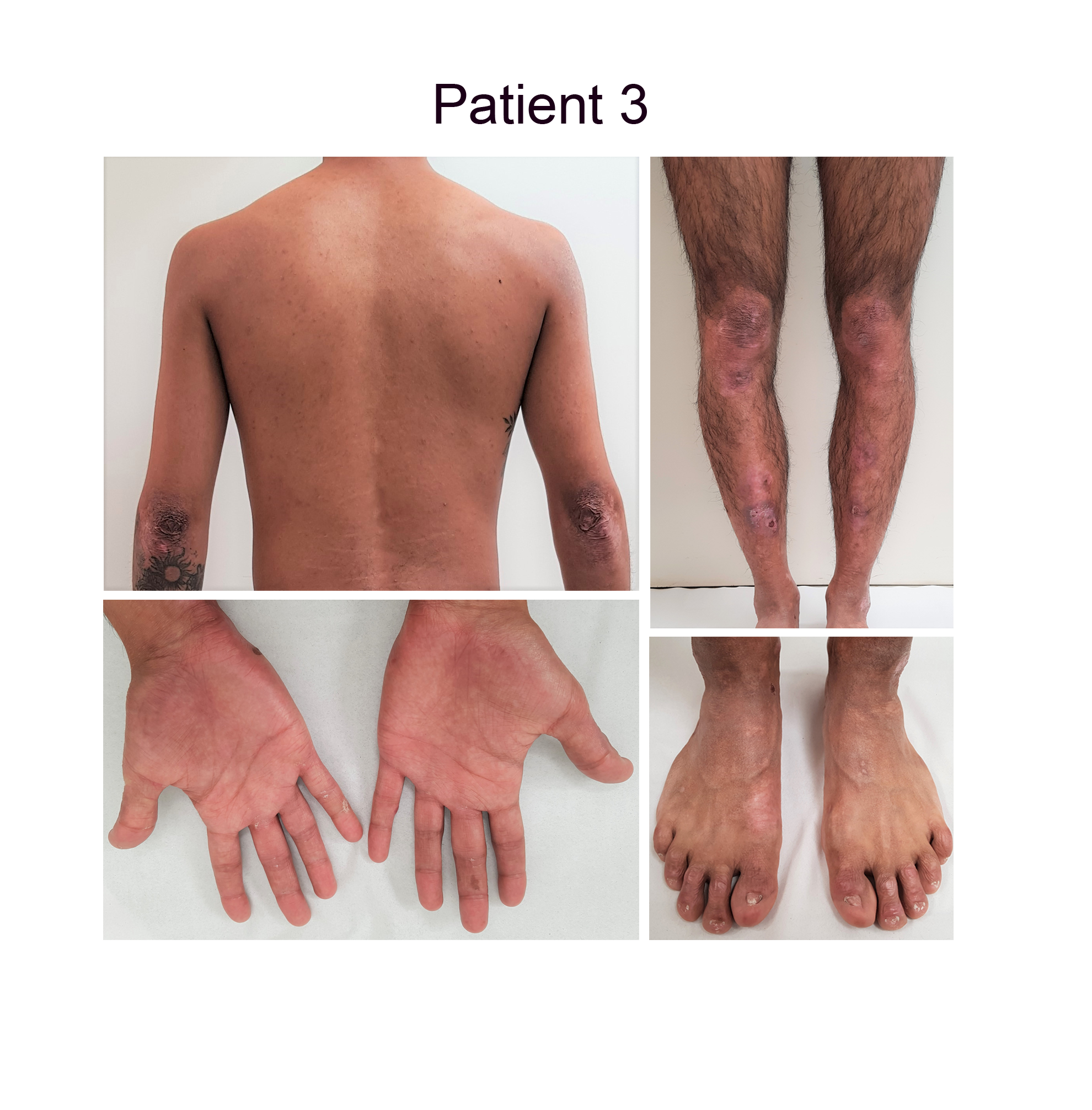

Supplement: Supplementary file 3 — Figure S3 [file 41431_2024_1717_MOESM3_ESM.tif]
