# Supplementary material for: Novel variants impairing Sp1 transcription factor binding in the COL7A1 promoter cause mild cases of recessive dystrophic epidermolysis bullosa
Source: Eur J Hum Genet. 2024 Dec 5;33(3):344–50. doi: 10.1038/s41431-024-01717-5 (PMC11894107; doi:10.1038/s41431-024-01717-5)
Supplement: Supplementary file 4 — Supplementary materials [file 41431_2024_1717_MOESM4_ESM.docx]

**SUPPLEMENTARY MATERIALS**

**Figure S1:** Clinical illustration of patient 1 presenting with intermediate RDEB with limited digital fusion of fingers, blisters with prominence over hands, feet, elbows and knees, and no mucous membrane involvement

**Figure S2:** Clinical illustration of patient 2 presenting localized RDEB with skin blistering limited to hands and feet, occasionally to pretibial skin, with milia and loss or dystrophic finger and toe nails.

**Figure S3:** Clinical illustration of patient 3 presenting with intermediate RDEB with predominantly blistering on hands, feet, elbows and knees, loss of finger and toe nails, no mucosal lesion and no digital fusion.
